# The role of Nrf2 in the pathogenesis and treatment of ulcerative colitis

**DOI:** 10.3389/fimmu.2023.1200111

**Published:** 2023-06-05

**Authors:** Shuai Peng, Lei Shen, Xiaoyun Yu, Li Zhang, Ke Xu, Yuan Xia, Lanlan Zha, Jing Wu, Hesheng Luo

**Affiliations:** ^1^ Department of Gastroenterology, Renmin Hospital of Wuhan University, Wuhan, China; ^2^ Hubei Key Laboratory of Digestive Diseases, Wuhan, China; ^3^ Department of Gastroenterology, Union Hospital, Tongji Medical College, Huazhong University of Science and Technology, Wuhan, China; ^4^ Department of Neurology, Renmin Hospital of Wuhan University, Wuhan, China

**Keywords:** Nrf2, ulcerative colitis, oxidative stress, intestinal fibrosis, colorectal cancer

## Abstract

Ulcerative colitis (UC) is a chronic inflammatory bowel disease involving mainly the colorectal mucosa and submucosa, the incidence of which has been on the rise in recent years. Nuclear factor erythroid 2-related factor 2 (Nrf2), known for its key function as a transcription factor, is pivotal in inducing antioxidant stress and regulating inflammatory responses. Numerous investigations have demonstrated the involvement of the Nrf2 pathway in maintaining the development and normal function of the intestine, the development of UC, and UC-related intestinal fibrosis and carcinogenesis; meanwhile, therapeutic agents targeting the Nrf2 pathway have been widely investigated. This paper reviews the research progress of the Nrf2 signaling pathway in UC.

## 1 Introduction

Ulcerative colitis (UC) is a nonspecific, chronic, relapsing inflammatory disorder mainly involving the mucosa and submucosa of the colorectum. The occurrence and frequency of UC have witnessed a continuous escalation in the progressive timeline of recent years (1). The disease has a long course and a wide range of lesions. Furthermore, it is essential to note that the principal clinical indications linked to this ailment encompass frequent diarrhea, excruciating abdominal pains, viscid mucus discharge, excrement that is conspicuously blood-stained, and even additional debilitating symptoms that cause a tremendous amount of distress and utterly corrode the quality of an individual’s life (2). It is also paramount to comprehend that this unfortunate medical predicament might lead to a sequence of menacing and detestable complications, such as damage to the intestinal tract resulting in the development of intestinal fibrosis and ultimately culminating in the malignant and life-threatening ailment commonly known as colorectal cancer (1).

The underlying causes of ulcerative colitis are murky and complicated. Yet the evidence is mounting that oxidative stress and inflammation are also closely related, except in genetics, intestinal flora, host immune system, and environmental factors (2). An accumulating corpus of empirical data has demonstrated the significant contribution of oxidative stress in inciting the inflammatory response that precipitates the onset of UC. Its multifaceted effects have impinged upon the disorder’s progression (3). Dysregulation of the immune system and pronounced inflammatory response events elevate reactive oxygen species (ROS) levels within the organism, thus perturbing redox balance and precipitating oxidative stress. The ensuing upsurge in ROS levels and consequential enhancement of oxidative stress indices result in deleterious cumulative damage to the fundamental biomolecules. Additionally, the imbalance between oxidative and antioxidant systems facilitates the activation of oxidative stress-associated pathways, which mediate cellular senescence, apoptosis, and necrosis (4). Nuclear factor erythroid 2-related factor 2 (Nrf2), a crucial transcriptional regulator involved in redox homeostasis, exerts a pivotal role in facilitating antioxidant responses within the organism (5). The Keap1/Nrf2 signaling pathway is constituted by the principal modulator Nrf2 and its counteractive inhibitor Kelch-like ECH-associated protein 1 (Keap1). This signaling pathway has been confirmed to exert a safeguarding influence on animal models and individuals with ulcerative colitis. In this regard, the Keap1/Nrf2 signaling pathway is vital as an antioxidant defense mechanism (3).

This paper aims to provide a comprehensive overview of Nrf2, including its physiological configuration and function, its involvement in intestinal maintenance and development, and its research progress in addressing ulcerative colitis and related complications. Among multiple aspects of Nrf2, we also focus on the therapeutic applications of modulating the Keap1/Nrf2 pathway in ulcerative colitis.

## 2 The physiological structure and function of Nrf2

### 2.1 The physiological structure of Nrf2

Nrf2 was initially cloned from the human leukemia cell line (K562) and identified as a Cap-n-collar (CNC) alkaline leucine zipper transcription factor family member (6). Nrf2 consists of seven Neh domains (Nrf2-ECH homology), each with a different function. The Neh1 domain is characterized by a remarkably conserved basic region-leucine zipper (bZIP) architecture. The Neh2 region mediates interactions with Keap1 through DLG and ETGE motifs. The carboxyl terminus of Neh3 has been found to facilitate the regulation of the antioxidant response element (ARE)-mediated transcription through its association with the chromo ATPase/helicase DNA binding protein (CHD6) (7). Neh4 and Neh5 regions, on the other hand, are instrumental in initiating downstream gene transcription, which is crucial for the transactivation of Nrf2. Notably, the regulatory region of Neh6 is characterized by the prevalence of serine residues and is responsible for the regulation of Nrf2 degradation via a mechanism independent of KEAP1. Furthermore, retinoic acid X receptor alpha (RXRα) has been reported to reduce the cytoprotective effect of Nrf2 by directly binding to the Neh7 domain (8).

**Figure 1:**
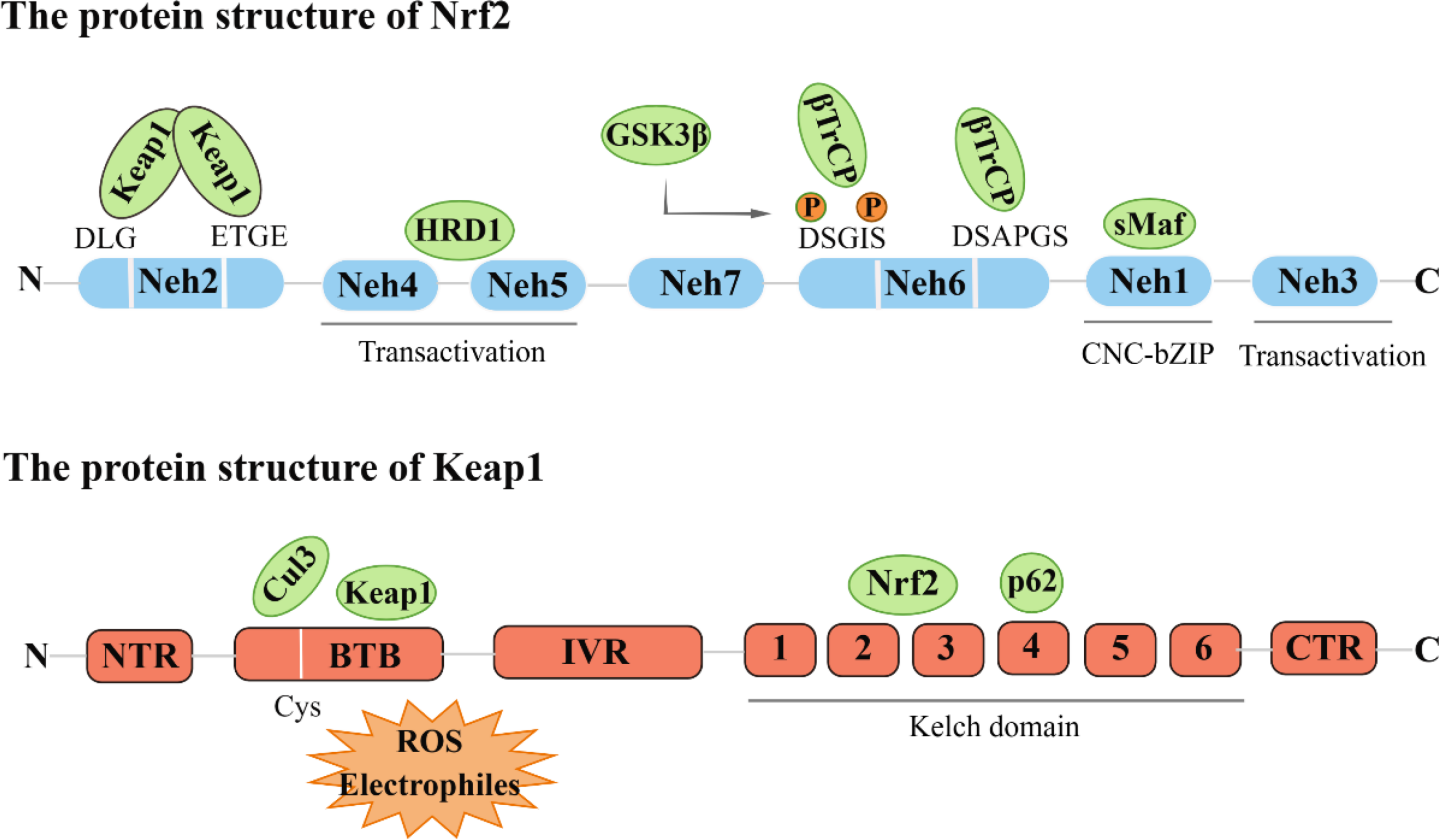
The protein structure of Nrf2 and Keap1.

Keap1 acts as a substrate adaptor protein for the E3 ubiquitin ligase complex, which comprises Cullin3 (Cul3) and Rbx1 to form a functional E3 ubiquitin ligase complex (Keap1-Cul3-E3). This complex plays a crucial role in regulating the activity of Nrf2 (9). Keap1 contains five domains, namely N-terminal region (NTR), intervention region (IVR), Broad complex, Tramtrack and Bric-à-Brac region (BTB), diglycine repeat region (DGR), and C-terminal domain C (CTR). The DGR region, the Kelch region, is the Neh2 junction region of Keap1 and Nrf2 (10) (**Figure 1**).

### 2.2 Triggers for activation of the Nrf2 signaling pathway

#### 2.2.1 Keap1-dependent activation of Nrf2 signaling pathway

Upon Nrf2 entering the nucleus, bZIP cooperates with small Maf proteins to form a heterodimer, enabling Nrf2 to recognize, bind to antioxidant response element (ARE), and initiate downstream related gene transcription (11). Under typical physiological circumstances, the BTB domain within the Keap1 protein interacts with the Cul3 protein. In contrast, the DGR domain targets various lysine residues in the Neh2 domain of Nrf2, thereby facilitating its ubiquitination. Consequently, the ubiquitinated Nrf2 undergoes degradation via the proteasome pathway (12). However, upon exposure to oxidative stress, particular cysteine residues within Keap1 undergo modification, which results in a structural alteration of the Keap1-Cul3-E3 ubiquitin ligase complex. This alteration disrupts the ubiquitination process of Nrf2, enabling its translocation into the nucleus. Subsequently, the nuclear Nrf2 forms a complex with sMaf proteins and binds to the ARE region. This binding event initiates a coordinated activation program that induces the expression of multiple cytoprotective genes, ultimately enhancing cellular defense mechanisms (13).

**Figure 2:**
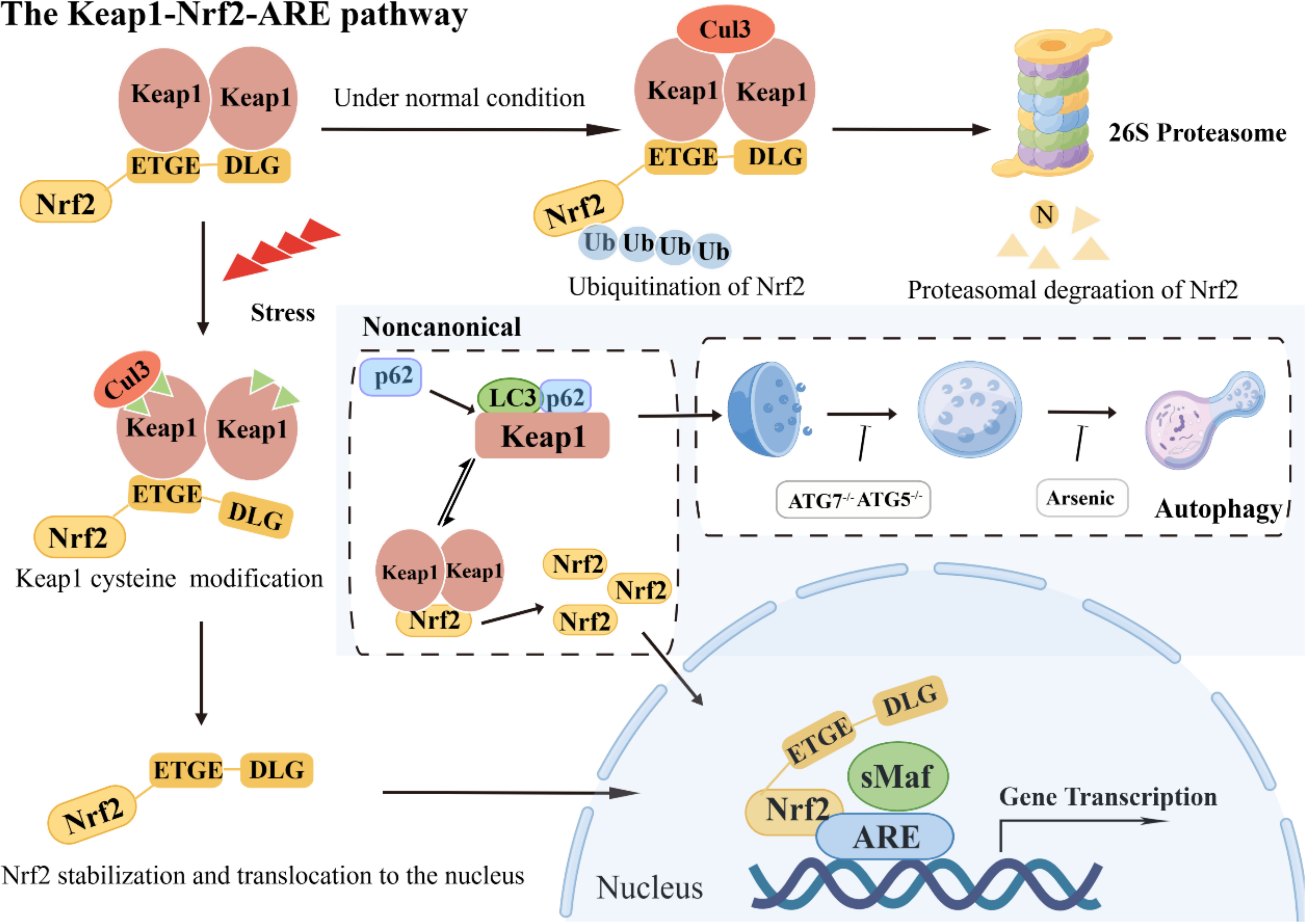
The Keap1-Nrf2-ARE pathway: classical and noncanonical pathways.

In addition to the Keap1/Nrf2 pathway, an atypical mechanism of Nrf2 activation, the autophagy-lysosome pathway, is driven by autophagy dysfunction, which also plays a crucial role in mediating oxidative stress (14, 15). The SQSTM1/p62 is a typical receptor for selective autophagy that degrades ubiquitinated substrates and plays a crucial role in regulating various signaling cascades, encompassing the Keap1/Nrf2 pathway (16). Dysregulation of autophagy processes culminates in the build-up of the autophagy-associated adaptor molecule SQSTM1/p62, consequently leading to the entrapment and subsequent functional impairment of various interacting proteins, including Keap1 (17). It has been reported that SQSTM1/p62 competes with Nrf2 for binding to Keap1, and this interaction can sequester Keap1 into autophagosomes, thereby preventing Keap1-mediated Nrf2 degradation and leading to Nrf2 pathway activation (18). Notably, the activation of Nrf2 must be tightly controlled. Over-accumulation or over-expression of Nrf2 has been reported to be detrimental and sometimes fatal (**Figure 2**).

#### 2.2.2 Keap1-independent activation of Nrf2 signaling pathway

Protein kinases are important regulators of many cellular processes through the phosphorylation of specific proteins. Recent studies have highlighted the crucial role of protein kinases in the modulation of Nrf2 activity. Among them, protein kinase C (PKC) has been shown to play a significant role in the activation and expression of Nrf2. Salvianolic acid B has been demonstrated to induce the expression of Nrf2, HO-1, and GCLC by activating the PI3K and PKC pathways, thus providing protection against APAP-induced liver injury (19). Similarly, protocatechualdehyde has been found to protect the liver from APAP-induced injury through the protein kinase Cϵ/Nrf2/HO-1 pathway and to alleviate cerebral ischemia-reperfusion-induced oxidative injury (20). Further investigations have revealed that the phosphorylation of Nrf2 at Ser-40 by PKC represents a key signaling event leading to the activation of ARE-mediated cellular antioxidant responses (21). Different isoforms of PKC may also play a role in mediating the phosphorylation of Nrf2 (22). Specifically, Chen et al. identified PKC-δ as the major PKC isoform responsible for the phosphorylation of Nrf2 Ser40, whereas PKC α and β activate Nrf2 at different time points, namely early and late stages, respectively (23).

Recent studies have highlighted the beneficial impact of AMP-activated protein kinase (AMPK) on the activation of Nrf2 and the subsequent induction of antioxidant enzymes in response to oxidative stress (24). AMPK has been found to trigger the expression of SOD and HO-1 via the Nrf2/ARE signaling pathway, leading to enhanced cellular antioxidant capacity and improved cell survival (25). Furthermore, AMPK has been shown to stimulate the nuclear accumulation of Nrf2 through phosphorylation at serine 550 (26). However, the interplay between AMPK and Nrf2 transcription warrants further investigation to better understand the underlying mechanisms of this signaling pathway.

Moreover, three Mitogen-activated protein kinases (MAPKs) have been identified as playing a role in the modulation of Nrf2 activity. Extracellular-signal-regulated kinases 1 and 2 (ERK1/2) are involved in both cell proliferation and defense mechanisms. Senkyunolide I has been found to protect the rat brain against focal cerebral ischemia-reperfusion injury by upregulating p-Erk1/2, Nrf2/HO-1, and inhibiting caspase 3 (27). Similarly, Gastrodin has been shown to safeguard the midbrain from oxidative stress in mice by blocking the ERK1/2-Nrf2 pathway (28). In addition, c-Jun N-terminal kinases (JNKs) and p38 MAPK are crucial mediators of oxidative stress reduction. Research indicates that Protocatechuic acid induces the expression of antioxidant/detoxification enzymes via JNK-mediated Nrf2 activation in mouse macrophages (29). Moreover, Ma et al. have identified p38 MAPK/Nrf2 signaling as a critical molecular network responsible for the development of temozolomide resistance in gliomas (30). Furthermore, Diallyl trisulfide has been shown to inhibit tumor growth by suppressing Nrf2/Akt activity and activating p38/JNK signaling (31). Meanwhile, Qi et al. have demonstrated that the activation and interplay between PI3K/Akt and Nrf2/HO-1 signaling pathways may play a role in regulating the hormesis of Z-ligustilide in PC12 cells subjected to oxygen and glucose deprivation (32). Similarly, reactive oxygen species and PI3K/Akt signaling have been shown to be key factors driving Nrf2-mediated heme oxygenase-1 expression in sulforaphane-treated human mesothelioma MSTO-211H cells (33). In conclusion, further investigation is necessary to gain a deeper understanding of the Keap1-independent Nrf2 pathway and its activation.

## 3 The role of Nrf2 in intestine development

The transcriptional activity of Nrf2 is essential for maintaining normal intestinal architecture in mice (34). The mouse intestine begins to establish around 9.5 days after conception. During the 9.5-14.5 days of embryonic development, pure epithelial cells transform into endodermal tubes, and the length and circumference of the intestine gradually increase (35). Subsequently, differentiated cells of absorptive and secretory lineages appeared from day 14.5, and epithelial remodeling and transient villi appeared in the gut on day 15. During 14.5-21 days of embryonic development, intestinal cells proliferate, and crypts and stem cell nests gradually form (36). Most colonic structures and cellular aggregates in adults exist prenatally, with fully developed colonic crypts manifested within 12-15 days following birth (35).

Intestinal development is mainly driven by the coordination between Notch and Wnt signaling pathways, and it is worth noting that Nrf2 can affect the activation of these signaling pathways (37). Studies have validated the significance of intercommunication between the Nrf2 and Notch pathways in regulating gastrointestinal tract maturation (38). Research in this area has shown that the proximal region of the promoter of the Notch downstream effector Math1 gene in mice has a functional ARE sequence, and the activation of Nrf2 signaling in the intestinal epithelium can lead to intestinal elongation and extension through negative transcriptional regulation of the Notch downstream effector Math1 (39). Meanwhile, Nrf2 knockout mouse embryonic fibroblasts decreased the expression of Notch-1 and its related gene signaling (40). There is a delicate balance between the Wnt and Nrf2 signaling pathways. A study confirmed that β-catenin in the Wnt pathway could activate the Nrf2 pathway to a certain extent, and Nrf2 could strongly inhibit β-catenin. Moreover, β-TrCP1 binds to β-catenin to weaken the inhibition effect of Nrf2 on β-catenin (41).

Twenty-five years ago, Chen et al. showed that the Nrf2 gene is highly expressed on the luminal side of the intestine during pregnancy in mice. In addition, they also observed substantial variations in Nrf2 mRNA levels across different organs and gestational days (42). A recent study has shown that Nrf2 levels continue to rise in the hindgut from 14.5 to 18.5 days of embryonic development, while Nrf2 levels decrease in other tissues, such as the lung or heart suggesting that Nrf2 plays a crucial role in intestine development (34). Nrf2 transcriptional deletion resulted in marked elongation of the colon, altered crypt distribution, enlarged goblet cells, and markedly elevated mucin levels (43). Thus, Nrf2 transcriptional activity constitutes an integral regulatory mechanism in the formation of the gastrointestinal tract, modulating the proliferation and differentiation of hindgut cells throughout varied embryonic stages.

## 4 The role of Nrf2 in ulcerative colitis

### 4.1 Nrf2 attenuates intestinal inflammation and damage by controlling oxidative stress

Oxidative stress results from dissonance amidst the production and clearance of ROS and reactive nitrogen species (RNS) following the body’s exposure to diverse injurious stimuli, which causes the body’s oxidation and oxidation antioxidant system imbalance. The antioxidant defense function is weakened, resulting in various pathological changes (44). Under physiological conditions, oxidation and anti-oxidation maintain a dynamic balance in the body. However, when inflammation occurs in the body, this balance is broken. Oxygen free radicals attack their tissues, participate in and generate inflammatory mediators through lipid peroxidation, and activate the inflammatory response (45).

Impairment of the antioxidant defense machinery in the gastrointestinal tract has been implicated in UC etiology. Infiltration of inflammatory cells exacerbates oxidative stress via the upregulation of ROS synthesis in the immune cells (46). Insurmountable ROS release, coupled with the persistent accumulation of oxidative stress, frequently culminates in DNA damage, protein oxidation, and lipid peroxidation, ultimately causing intestinal tissue damage, debilitating the immune system, and precipitating an array of severe pathologies, including UC (47). When UC occurs due to the inflammatory response, the activity of gut-derived vasoconstrictors is enhanced, resulting in intestinal ischemia. At the same time, inflammatory cells in the intestinal mucosa, such as neutrophils and macrophages, enter the intestinal tract from the blood circulation (48). In the damaged part, under the action of cell membrane reduced coenzyme II and NADPH oxidase, a large number of reactive oxygen radicals such as superoxide anion (H:0), hydrogen peroxide (H2O2), hydroxyl radical (H01), NO free radicals and lipid peroxides (LPO), thereby aggravating intestinal mucosal damage (47, 49).

Cells have evolved a complex protective system to defend against the damage mentioned above, and the Keap1/Nrf2 pathway is the foremost defense mechanism for counteracting oxidative stress (13). The initial research investigating the alleged involvement of Nrf2 in UC was reported first by Arisawa et al. in 2008. They identified that the -686*-684 genotype of the Nrf2 gene was significantly correlated with UC incidence among the Japanese population and closely associated with the chronic persistent phenotype (50). Recent research has established that the expression level of Nrf2 in individuals with UC is lower than in healthy cohorts. Nonetheless, some studies have also revealed a marked increase in the expression level of Nrf2 in the mucosa of the inflammatory intestinal tract among UC patients compared to controls (51, 52). Moreover, Milad’s research demonstrated that the phosphorylated form of Nrf2 was expressed at a significantly higher level in individuals diagnosed with moderate and severe UC than in healthy controls. Conversely, the expression level of non-phosphorylated Nrf2 was diminished in moderate to severe UC patients compared to healthy cohorts. These observations suggest that the altered levels of Nrf2 in UC patients may be influenced by the disease’s progression or the form of Nrf2 expression (51).

Over the years, related studies on the involvement of the Keap1/Nrf2 axis in the process of UC have found that mice lacking Nrf2 exhibit heightened vulnerability to dextran sodium sulfate (DSS)-induced colitis and an increased susceptibility to colorectal cancer (53–55). Furthermore, compared with wild-type mice, the levels of pro-inflammatory cytokines and lipid peroxidation in the colon of Nrf2 knockout mice treated with DSS were significantly increased, and the expression levels of antioxidant enzymes were decreased (56). Moreover, multiple genetic mutations on Nrf2 have been linked to increased susceptibility and progression of DSS-induced colitis in mice (57, 58). Considering these discoveries, modulation of the Keap1/Nrf2 signaling pathway could represent a promising avenue for managing UC.

In UC, the Keap1/Nrf2 pathway can reduce intestinal inflammation and injury by controlling oxidative stress and play an essential role in protecting intestinal integrity, mainly by regulating inflammatory mediators and inducing the production of antioxidant enzymes (59, 60). Oxidative stress provokes Nrf2 to translocate to the nucleus, where it mediates the transcription of a diverse suite of antioxidant genes, thereby conferring cell protection against the damage induced by oxidative stress (61). In addition, various antioxidant enzymes in the body, such as superoxide dismutase (SOD), glutathione (GSH), and other activations, establish an endogenous defense system against intestinal oxidative stress, protecting the intestinal mucosa from harmful stimuli (62). Currently, most studies focus on regulating inflammatory mediators, inducing the production of antioxidant enzymes, and regulating autophagy by activating Nrf2, thereby reducing oxidative stress damage caused by aggravated ROS and alleviating pathological inflammatory responses (63–66).

In addition, activation of Nrf2 can balance cellular homeostasis, activating a series of signaling pathways targeting inflammation, such as the NF-κB pathway. Numerous studies have shown an interaction between the Keap1/Nrf2 and NF-κB pathways (67, 68). First, sMaf (MafK) can positively regulate NF-κB activity by enhancing the Nrf2 transcriptional coactivator CBP-mediated acetylation of NF-κB p65, suggesting that Nrf2 may indirectly regulate NF-κB activity by inhibiting MafK. Second, Keap1 can inhibit the activation of NF-ĸB by inhibiting the ubiquitination degradation of IKKβ. Third, the inflammatory response can inhibit NF-κB activity by inducing inflammatory mediators and subsequently reacting with Keap1 to activate the expression of Nrf2 (69, 70). In conclusion, activation of Nrf2 in the gut can inhibit inflammatory pathways or reduce the overreaction of oxidative stress, thereby alleviating intestinal damage and inflammation.

However, we still need to control the expression of Nrf2 strictly. Recently, in a study of transgenic mice constitutively expressing active Nrf2 (caNrf2 mice), Gerstgrer et al. found that symptoms of acute colitis induced by DSS were exacerbated after constitutive Nrf2 expression but not worsened chronic colonic mucosal inflammation (71). The above phenomenon suggests that the redox balance in the body needs to be strictly regulated. Otherwise, the double-edged sword effect is prone to occur. Meanwhile, further extensive studies are therefore imperative for an enhanced understanding of the complex interplay between the Keap1/Nrf2 signaling network and oxidative stress as well as inflammation in the intestine.

### 4.2 Nrf2 facilitates the maintenance of the intestinal epithelial barrier

The intestine is an important digestive organ of the human body and the largest immune organ of the body. The intestinal epithelium forms a tightly regulated intestinal barrier between the external environment and the body, which can prevent the invasion of harmful substances such as pathogenic bacteria and toxins. It is indispensable in maintaining homeostasis and body health (72, 73).

The intestinal mucosal barrier is a multifaceted structure comprising the surface mucous layer, epithelial cell layer, and mucosal basal layer, alongside the biological barrier facilitated by the resident microflora of the gut. Furthermore, it encompasses a chemical barrier consisting of a range of digestive enzymatic secretions, lysozymes, mucopolysaccharides, glycoproteins, and glycolipids produced by the intestine, an immune barrier that is formed by intestinal associated lymphoid tissue (GALT) and secretory immunoglobulin A (sIgA), and a mechanical barrier consisting of intact intestinal mucosal epithelial cells and intercellular junctions (74–76).

The pivotal role of the intestinal mucosal barrier is attributed to its mechanical barrier, in which epithelial cells and their intercellular junctions are the structural basis for maintaining intestinal epithelial selective permeability and barrier function. It is the key to resisting the invasion of extraintestinal harmful substances or pathogens into the intestinal mucosa (77, 78). The junction between intestinal epithelial cells is the core part of the mechanical barrier. It is controlled by the myosin light chain (MIC), including tight junctions, gap junctions, adhesion junctions, and desmosome junctions, especially tight junctions, mainly composed of occlusal junctions. The constituents of this barrier include members of the tight junction protein group, such as occludin, claudin, and cadherin, as well as the Zonula Occludens (ZO) family (79, 80). The tight junctions between intact intestinal epithelial cells can prevent intestinal bacteria, toxins, and antigens from entering the lamina propria, prevent the activation of lamina propria immune cells, and induce abnormal intestinal immune responses (81, 82).

A key pathological event in UC is intestinal barrier dysfunction. The destruction of the intestinal barrier will increase intestinal permeability, and intestinal pathogenic bacteria and pathogens will further invade the intestinal mucosa, thereby exacerbating inflammatory cell infiltration and damage, forming a vicious circle, which eventually leads to damage to the intestinal mucosa and ulcer formation (83, 84). Current research confirms the indispensable involvement of Nrf2 in preventing UC. Activation of Nrf2 in animal models of UC exerts a regulatory effect on the expression of tight junction proteins located in the intestinal epithelium, specifically Zonula Occludens-1 (ZO-1) and claudin, thus safeguarding the integrity of the gut barrier (85–87). In DSS-induced mouse colitis models, tight junction protein expression was significantly lower than in the control group, resulting in increased intestinal permeability (88, 89). In the LPS-induced intestinal barrier damage model, the mitochondria-targeted antioxidant MitoQ can prevent intestinal barrier damage by upregulating the expression of Nrf2 downstream regulatory genes. The mechanism may involve activating the Keap1/Nrf2/ARE signaling pathway and inhibiting oxidative stress (90). Concurrently, the study revealed that Nrf2 confers a safeguarding effect in a traumatic brain injury-induced intestinal mucosal injury model. In contrast to wild-type mice, Nrf2-deficient mice demonstrated enhanced susceptibility to traumatic brain injury-induced intestinal inflammation, characterized by elevated intestinal permeability and augmented plasma endotoxin levels, exacerbating the decline in intestinal barrier function (91). Another study in the same model confirmed that ERK/Nrf2/HO-1-mediated stimulation of mitophagy ameliorates intestinal mucosal impairment and barrier dysfunction (92). Furthermore, Nrf2 activation is purported to enhance the protection of tight junction proteins by negating apoptosis of intestinal epithelial cells while concurrently instigating autophagy. In addition, in a study of reflux esophagitis, Nrf2 was found to bind to the promoter of claudin-4 and increase its expression but not to claudin-1. Nrf2 deficiency leads to mitochondrial dysfunction, downregulating claudin-4 expression and ultimately leading to tight junction damage in the esophageal epithelium (93). Further research has demonstrated that the inducement of the Keap1/Nrf2 pathway impacts the regulation of tight junction proteins and governs the regenerative processes of intestinal stem cells, thereby promoting intestinal homeostasis (94, 95).

The intestinal mucus barrier also plays an integral role in maintaining gut health (96). Intestinal mucus is a high-molecular glycoprotein mucus layer secreted by goblet cells, which forms a functional barrier between intestinal microbes and the intestinal epithelium, providing defense by hindering the direct contact of bacteria and harmful substances to the intestinal epithelium (97). Research has documented that diminished levels of intestinal mucus can hinder nutrient absorption through the intestinal mucosa while simultaneously triggering secretion of water and electrolytes within the intestinal lumen. At the same time, plasma-like fluid penetrates further into the lumen, increasing the volume of fluid and the permeation load in the lumen and causing diarrhea (98). Moreover, the study found that Low molecular Seleno-amino polysaccharide (LSA) can protect the intestinal mucosal barrier in rats by activating the Nrf2 pathway and mitigating the anomalous alterations of MUC2 (99). In addition, Singh and his team have found that gut microbial metabolism enhances the integrity of the intestinal barrier through the Nrf2 pathway (59). To conclude, the activation of Nrf2 aids in preserving the integrity of the intestinal epithelial barrier, while further investigations are required to elucidate the underlying mechanism involved.

### 4.3 Nrf2 is the regulator of intestinal immunity

The abnormal intestinal mucosal immune system is one of the main reasons for the development of UC. Meanwhile, initiating intestinal mucosal immunity and ensuing inflammation hinges upon activating effector T cells. In response to a plethora of stimuli, an array of effector T cell subsets emerge from precursor naive T cells, including Th1, Th2, Th17, and Treg cells (100, 101).

In recent years, in addition to the anti-inflammatory mentioned above and oxidative stress control effects of Nrf2, numerous research endeavors have additionally evidenced that Nrf2 activation holds the potential to modulate the Th1/Th2 equilibrium selectively. In 2012, Rockwell and his team’s research found that the induction of Nrf2 can preferentially direct CD4+ T cells toward Th2 differentiation. Specifically, the activation of Nrf2 by tBHQ, a food preservative, has been observed to hinder the production of the Th1 cytokine IFN-γ and simultaneously encourage the generation of Th2 cytokines such as IL-4, IL-5, and IL-13 (102). Three years later, the team found that the Nrf2 activator tBHQ inhibited the production of IL-2 and IFN-γ in activated CD4^+^ T cells (103). Five years later, the team continued to investigate the effects of Nrf2 activation on the initial events that follow T-cell activation. The results showed that different Nrf2 activators (tBHQ and CDDO-IM) had different effects on early T cell differentiation, and the activation of some cytokines did not depend on the Nrf2 pathway. Although Nrf2 inhibited the expression of early TNF-α and IFN-γ after activation, it was observed to facilitate the generation of IL-2. It displayed no discernible effect on the induction of CD25 and CD69. IL-2 serves as a growth factor for T cells that promotes the development of Th2 cells and is critical for the differentiation and function of Treg cells (104). Other recent studies on tBHQ also confirmed that tBHQ attenuated 5-fluorouracil-induced intestinal epithelial cell injury by activating Nrf2 (105). Moreover, tBHQ induced an Nrf2-dependent increase in IgM secretion by LPS-stimulated B cells (106). Meanwhile, studies have shown that Nrf2 can regulate the IL-22 response in CD4^+^ T cells through the AhR pathway (107). This suggests that activation of Nrf2 has a relevant role in regulating both T and B cells.

**Figure 3:**
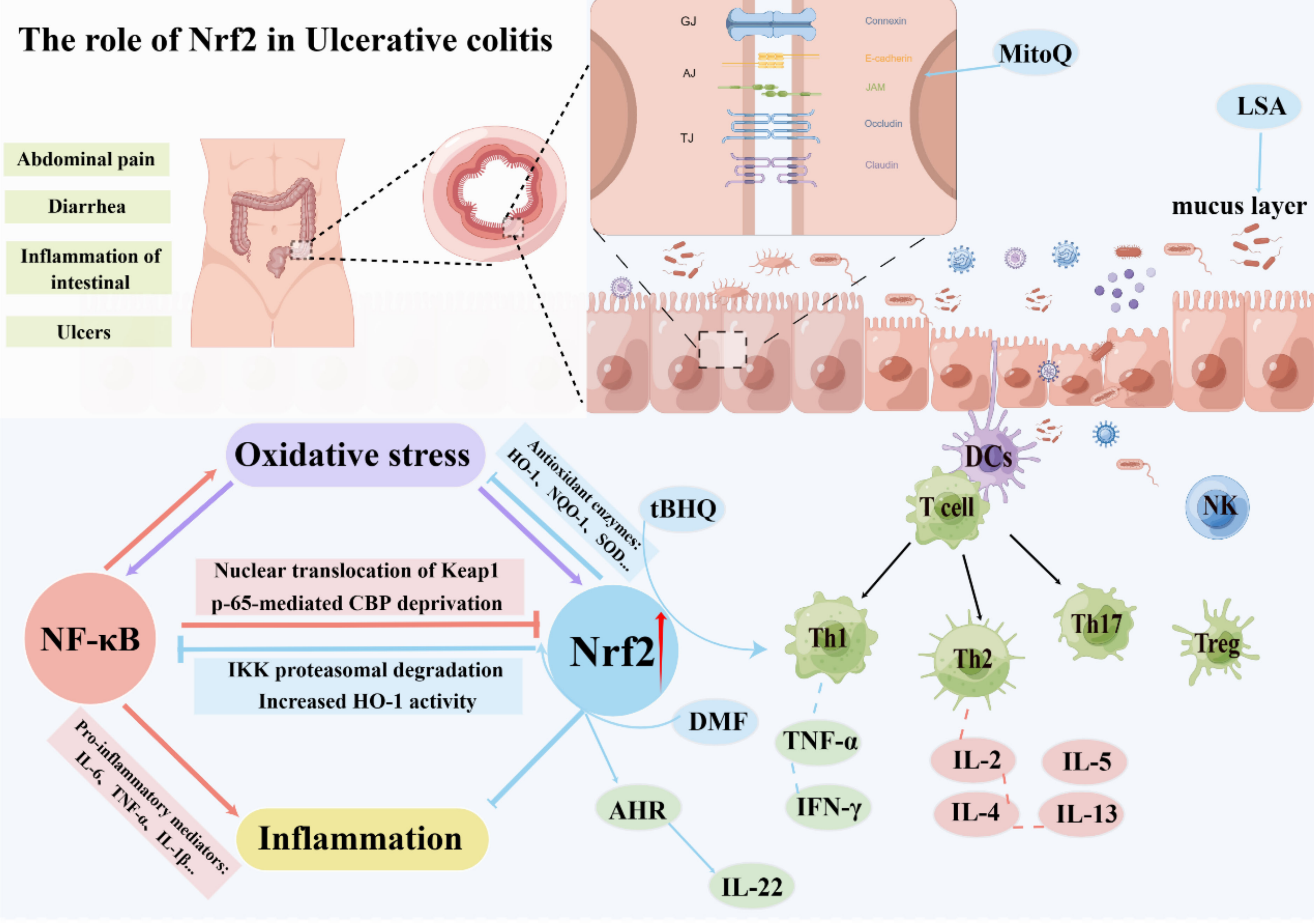
The role of Nrf2 in ulcerative colitis.

Another study focused on dimethyl fumarate (DMF). This Nrf2 activator demonstrated that DMF could reduce the inflammatory response in experimental colitis, mainly due to the activation of Nrf2 and its downstream antioxidant genes expression after administration of DMF and simultaneously inhibit the NF-κB signaling (108). A research investigation exploring acute graft-versus-host disease (AGVHD) unveiled that activation of Nrf2 by DMF promoted the development of donor Treg cells and reduced the deleterious response of allogeneic T cells (109). Although the activation of Nrf2 modulates intestinal immunity to varying degrees, the exact mechanism has not been fully elucidated (**Figure 3**).

## 5 The role of Nrf2 in intestinal fibrosis in UC

The accumulation of excessive extracellular matrix caused by chronic inflammation is a characteristic feature of intestinal fibrosis, which is a prevalent complication found in patients with Crohn’s disease (CD) and has more recently been observed in those with UC (110). During UC, ECM accumulates in the mucosa and submucosa, leading to its thickening, which results in the shortening and stiffening of the colon (111). Furthermore, fibrosis in UC is notably intertwined with inflammation and the disruption of the epithelial layer, often attributed to damage inflicted on the tight junctions (112).

The abnormal accumulation of ECM characterized by fibrosis may be due to overproduction or reduced degradation of ECM. Despite recognizing the TGF-β1/SMADs signaling pathway as the chief driving force behind intestinal fibrosis, numerous pro- and anti-fibrotic endogenous factors, such as ROS and Nrf2, have been identified as interactors of this pathway (113, 114). Several studies have shown that Nrf2 exerts an anti-fibrotic effect in various organs and that this protective effect is closely related to the classical pathway of fibrosis, TGF-β1/SMADs (115–117).

TGF-β1 and its receptor are highly expressed in animal models of fibrous stenosis and intestinal fibrosis. They can signal to the downstream Smads family of proteins, promoting extracellular matrix protein deposition and fibroblast transformation, thereby accelerating fibrosis (118, 119). At the same time, TGF-β1 promotes the generation of ROS while concurrently obstructing the activity of antioxidant enzymes, thereby creating an asymmetry within the redox homeostasis system, while ROS is also an essential mediator in activating the TGF-β1/Smads pathway (120). Therefore, redox disorders caused by dysregulation of the Nrf2 pathway induce fibrosis through massive ROS production and activation of the TGF-β1/Smads pathway (113). Guan et al. showed that tBHQ reduced fibrosis in mice with chronic fibrous colitis and human intestinal fibroblasts by inhibiting the TGF-β1/Smads signaling pathway. At the same time, Nrf2^-^/^-^ promoted TGF-β1-induced intestinal fibroblast differentiation by pretreating human intestinal fibroblasts with tBHQ or siNrf2. Nrf2^-^/^-^ could promote TGF-β1-induced intestinal fibroblast differentiation (114). Thus, the inhibition of intestinal fibrosis by Nrf2 is accomplished through the ROS/TGF-β1/Smads pathway, which has been demonstrated in both *in vitro* and *in vivo*.

Parallel results were observed in CCD-18Co, a type of normal human colonic fibroblasts, following stimulation with TGF-β1. Suppression of Nrf2 amplified the expression of the TGF-β1/Smad signaling cascade in CCD-18Co fibroblasts (116, 121, 122). These findings highlight the potential of Nrf2 activation in constraining the TGF-β1/Smad signaling axis and its consequent alleviation of intestinal fibrosis. Among colon-derived CCD-18Co fibroblasts, Nrf2 equipped itself to tone down intestinal fibrosis by putting the brakes on the ROS-dependent TGF-β1 signaling pathway, eliciting ROS scavenging (114, 116, 123).

MMPs and TIMPs regulate the degradation of the extracellular matrix. An irregularity in the functioning of these specific enzymes results in the accumulation of extracellular matrix (ECM), thereby contributing to the development of fibrosis (124, 125). Among the myriad matrix metalloproteinases (MMPs), MMP7 is the most critical component within the intestinal context. Research has demonstrated that in human intestinal epithelial cells, the Nrf2/HO-1 axis effectively suppresses MMP7 activity. Reducing fibrosis by specifically inhibiting MMP7 through modulation of the Nrf2 signaling pathway may greatly benefit the treatment of IBD (113, 126). In addition, growing evidence elucidates the role of MMP-3 in IBD.MMP3 concentrations have been observed to increase in response to oxidative stress, and both its expression and activity exhibit augmentation in mice lacking Nrf2. Significantly, patients diagnosed with CD and UC exhibit elevated levels of MMP-3 (127, 128). Furthermore, research has highlighted that MMP3 concentrations are pivotal in determining the responsiveness to infliximab therapy among individuals diagnosed with IBD. Those who did not respond within one year had significantly higher serum MMP3 levels than controls (129). Furthermore, MMP3 possesses the discriminatory capacity for differentiating pediatric patients with UC from their healthy counterparts (130). The implications of MMP3 in regulating intestinal barrier functionality have been comprehensively examined and elucidated by Giuffrida et al. (131). The above findings reveal new therapeutic strategies to modulate Nrf2 signaling to re-establish cellular homeostasis in intestinal fibrosis.

## 6 Two-sidedness of Nrf2 in UC-associated colorectal cancer

Colorectal cancer (CRC) is a frequently occurring malignancy with a substantial global burden, characterized by elevated incidence and mortality rates. Part of it is caused by chronic colitis, called colitis-related colon cancer (2). Numerous epidemiological, experimental pathological, and clinical studies have shown that the prolonged presence of inflammatory bowel disease, especially chronic ulcerative colitis, can lead to malignant transformation into colon cancer and even promote the progression and early metastasis of colon cancer (132). Studies have reported that UC is a precancerous lesion of colorectal cancer and that the prevalence of CRC in patients with UC is two to six times higher than in the general population, with the incidence increasing with the number of years of diagnosis (132).

**Figure 4:**
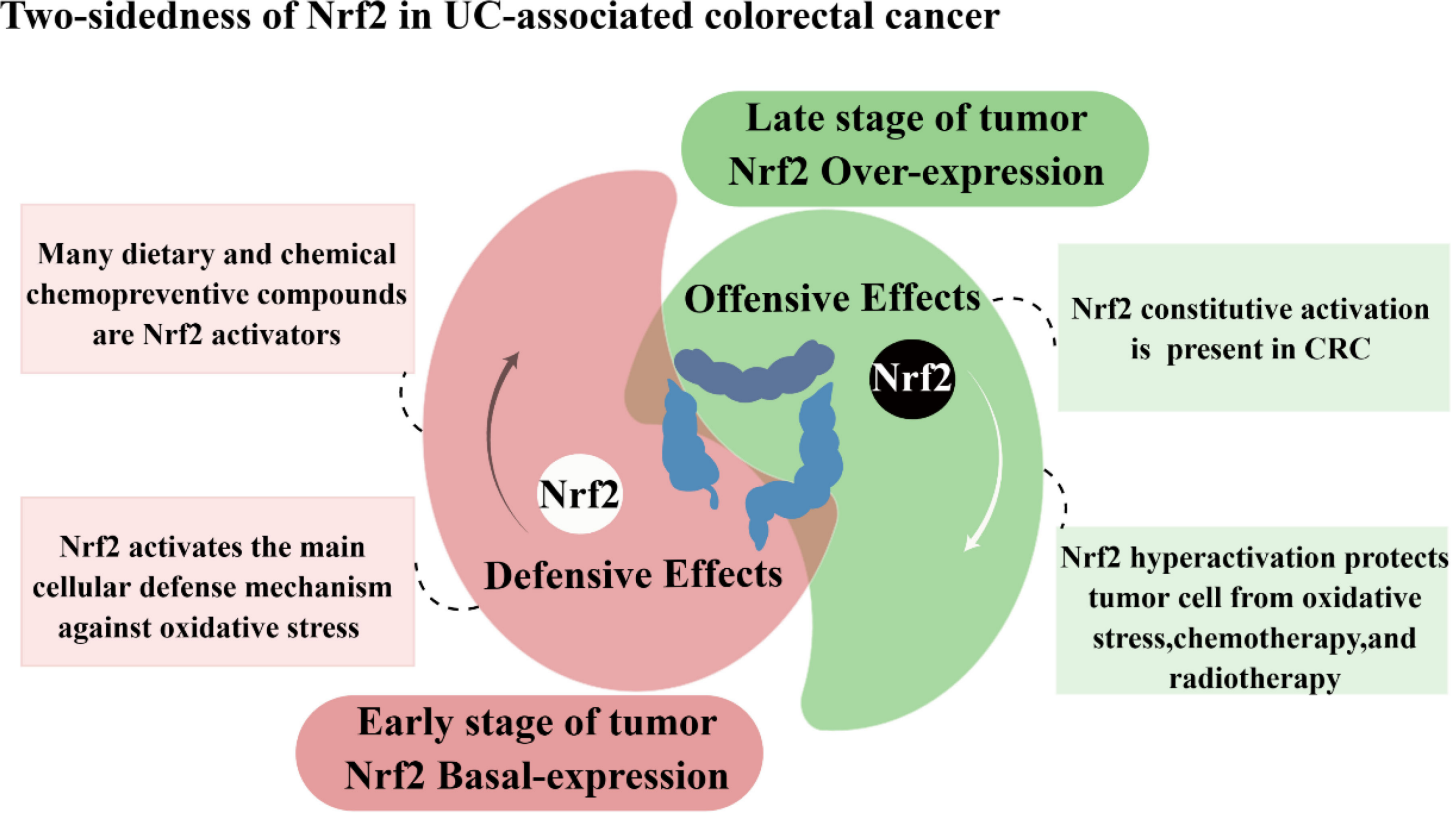
Two-sidedness of Nrf2 in UC-associated colorectal cancer.

As a classical pathway for cellular defense and survival signaling, the role of Nrf2 in tumors has been of great interest. In recent studies, evidence has emerged indicating that Nrf2 plays a role in the carcinogenic transformation of UC. In patients with active ulcerative colitis, a complex ROS-rich microenvironment consisting of cytokines, chemokines, and inflammatory cells in the inflammatory state of the intestine is a major factor in promoting the cancerous transformation of UC (3, 126). It is thought that Nrf2 plays a dual and controversial role in developing and progressing colorectal cancer associated with colitis (**Figure 4**).

Nrf2 has a protective role in the early stages of CRC development, and ensuring the appropriate basal regulation of Nrf2 is crucial for averting carcinogenesis in colon tissues. In colon cells, activation of Nrf2 protects colon cells from damage by reducing genotoxic damage produced by oxidative stress, thereby inhibiting colon cancer progression (133). Existing research suggests that when colon cells are subjected to dextran sodium sulfate, it leads to the induction of carcinogenesis in the colonic tissue. At the same time, the addition of CPUY192018, a Keap1-Nrf2-PPI signaling pathway inhibitor, activates Nrf2 to reduce the risk of conversion of ulcerative enteritis to colon cancer (134). In addition, digitalis flavonoids can activate Nrf2 via p38 MAPK, promote Nrf2 nuclear translocation, and stimulate the expression of downstream phase II detoxification enzymes. This process safeguards colonic cells from oxidative stress and subsequently decreases the occurrence of AOM-DSS-induced colorectal cancer (135). Conversely, the absence of Nrf2 regulation compromises the cellular capacity to withstand genotoxic and oxidative stress, resulting in a disrupted intestinal microenvironment, and normal colon cells become more susceptible to damage from oxidative stress and various genotoxic compounds. The study showed that Nrf2 knockout mice and mice normally expressing the Nrf2 gene was exposed to both the colitis-inducing agent DSS and the colon cancer-inducing agent AOM to observe the risk of colon cancer in both groups. The results showed that Nrf2 knockout mice exposed to DSS or AOM developed prolapse, rectal bleeding, and inflammation and increased the number of abnormal crypt foci, indicating that Nrf2 knockouts are more susceptible to colitis or colorectal cancer (53, 54, 136). In addition, the presence of single nucleotide polymorphisms (SNPs) within the promoter region of the Nrf2 gene is correlated with an elevated risk of neoplastic development, and Nrf2-silenced mice showed significantly higher levels of neoplastic damage and tumorigenicity than wild-type mice, as well as significantly higher levels of 8-hydroxydeoxyguanosine in the epithelial DNA of mice. This suggests that identifying molecules that maintain a constant state of Nrf2 could help prevent cancer development at an early stage (137).

Nrf2 has been implicated in the processes of cancer cell proliferation, metastasis, and resistance to radiotherapy during advanced tumor stages. Although Nrf2 was initially identified as a tumor suppressor owing to its protective function against exogenous and endogenous damage. However, mounting evidence suggests that the over-activation of the Nrf2 pathway facilitates tumor cell survival and protects them from oxidative stress and drug effects. Nrf2 is now thought to accelerate tumor progression, promote metastasis and participate in resistance to radiotherapy in advanced tumor stages.

Nrf2 overexpression can promote the development of colon cancer. One study found that colon cells were subjected to oxidative stress and produced excess ROS, which induced Nrf2 overexpression, leading to inflammation of colon tissue and promoting colon carcinogenesis (138). Arlt and colleagues discovered that heightened Nrf2 expression in colon cancer cells and augmented expression of proteasomal subunit proteins S5a/PSMD4 and α-5/PSMA5 increased proteasomal activity provided anti-apoptotic protection and adequate clearance of abnormal proteins in cancer cells and promoted colon carcinogenesis (139). Sebens et al. found that co-culture of M1-type macrophages with colonic epithelial cells activated Nrf2 expression and proteasome activity in colonic epithelial cells, rendering colon cells resistant to apoptosis, promoting inflammatory carcinogenesis, and increasing the risk of colon carcinogenesis (140, 141).

Nrf2 exhibits a strong association with tumor metastasis. Research has demonstrated that Nrf2 is potentially one of the indicative markers related to metastatic tumor processes. Nrf2 is highly expressed in highly invasive colorectal cancer tissues, and its expression is positively correlated with Duke’s stage and clinical prognosis, making it an important marker of colon cancer prognosis (142). Nrf2 regulates colon cancer metastasis by regulating vascular endothelial growth factor (VEGF) and its receptors. Neoangiogenesis is a critical factor contributing to the growth of colorectal cancer tissues. VEGF and its receptor display elevated activity levels during early and advanced (metastatic) stages of colon cancer. At the same time, VEGF family proteins and receptors trigger multiple signaling networks that cause endothelial cell survival, mitosis, and migration (143). One study found that inhibition of Nrf2 blocked the accumulation of HIF-1α in colon cancer cells under hypoxic conditions and inhibited the expression of VEGF and HIF-1α target genes while reducing the growth and angiogenesis of xenograft tumors in mice (144). Currently, anti-VEGF/VEGFR therapy is essential in treating metastatic colon cancer, improving progression-free survival (FPS) and overall survival (OS) in patients with colon cancer (145). Thus, the suppression of Nrf2 may potentially manifest inhibitory effects on the metastasis of colon cancer, thereby highlighting its prospective significance as a target for the treatment of metastatic colon cancer.

Nrf2 plays a vital role in colon cancer chemoresistance. Nrf2, an essential transcriptional regulator of oxidative stress, protects cells from oxidative stress and toxic damage from chemical drugs. However, many studies have reported that persistent overexpression of Nrf2 causes increased resistance of cancer cells to chemotherapeutic drugs, including adriamycin, etoposide, and cisplatin, suggesting that Nrf2 is an important transcription factor for tumor drug resistance (146). 5-Fluorouracil (5-FU) is the most commonly used chemotherapeutic agent in the treatment of colon cancer, but the development of resistance to 5-FU has dramatically reduced its clinical efficacy. The chemotherapeutic agent 5-FU exerts cytotoxic effects on colon cancer cells by inducing the generation of ROS, consequently causing oxidative damage and ensuing cell death. Nonetheless, tumor stem cells (CSCs), a subgroup of colon cancer cells, can counteract 5-FU-induced oxidative damage in colon cancer cells by producing an adaptive cellular response to ROS, closely related to Nrf2 activation, which causes upregulation of antioxidant enzymes and increases cancer cell resistance to 5-FU (147, 148). It was found that FoxO3 overexpression increased the sensitivity of colon cancer cells SW620 and HCT-8 to 5-FU and that the reversal of resistance of human colorectal cancer cells to 5-FU was demonstrated through the involvement of FoxO3 in the inhibition of the Nrf2/TR1 signaling pathway (149). In addition, Kang et al. found that the mechanism of 5-FU resistance in colon cancer was associated with epigenetic modifications such as DNA demethylation upregulating Nrf2 and HO-1 expression. By comparing the epigenetic changes associated with Nrf2 induction in the 5-FU-resistant colon cancer cell line SNUC5, it was concluded that Nrf2 expression, as well as its nuclear translocation and promoter binding, were markedly elevated in SNUC5/5-FUR cells compared to SNUC5 cells, and further Nrf2 or HO-1 knockdown mediated by siRNA considerably curtailed the proliferation of colon cancer cells both *in vitro* and *in vivo*, leading to heightened sensitivity to 5-FU (150). Cheng et al. demonstrated that cNrf2 exhibited resistance towards 5-FU and oxaliplatin, both *in vitro*, using the HCT116 cell line, and *in vivo*, employing the CRC animal model. This resistance was attributed to the PSMD4-mediated nuclear export of Nrf2, which ultimately activated the NF-κB/AKT/β-catenin cascade, further supporting these findings (151). In addition, cNrf2 and PSMD4-positive CRC patients had a higher rate of chemoresistance.

Nrf2 also plays a key role in promoting resistance to other chemotherapeutic agents in colon cancer. The significant abatement in SW480/Res cell migration and increased induction of apoptosis by oxaliplatin was observed upon inhibition of Nrf2 in colon cancer cells (152). In addition, Nrf2 reduced the sensitivity of NCM460 or Colo320 cells to TRAIL/etoposide by inducing proteasome activity, thereby reducing the apoptosis induced, and tissue immunostaining further confirmed the activation of Nrf2 in the colonic epithelium in the inflammatory region, as well as the increased proteasome expression (140)a. These studies suggest that Nrf2 has an important influence on the development of chemoresistance in colon cancer.

Nrf2 co-regulates CRC progression through interactions with other signaling pathways. In CRC, Nrf2 enhances NF-κB transcriptional activity, which is strongly associated with CRC cell invasion; positive and negative regulation of NF-κB and Nrf2 signaling pathways coexist, which may be closely linked to cell type and tissue microenvironment (153). At the same time, it has been found that Keap1 mutations lead to impairment of the Nrf2-Keap1-ARE signaling pathway, affecting its binding to Nrf2, causing a large accumulation of Nrf2, and increasing the resistance of tumor cells. In malignant tumors, the incidence of Keap1 loss of function is high. Keap1 mutations affect the inhibitory activity of Keap1 on Nrf2, and Keap1 loss of function enhances the survival of tumor cells (154). DeNicola et al. found that Nrf2 transcription was significantly increased in primary mouse cells following the expression of Kras, BRaf, and myc endogenous oncogenic alleles. The upregulation of Nrf2 target genes and the augmented stability of Nrf2 engendered by somatic mutations in both Nrf2 and Keap1 might serve as a mechanism for the increased expression of Nrf2 during tumorigenesis and progression (155). In addition, overexpression of Nrf2 in colon cancer cells could promote colon cancer progression through ERK and AKT signaling pathways (156).

## 7 The therapeutic potential of modulation of the Keap1-Nrf2 pathway in UC

Nrf2, a crucial transcription factor responsible for regulating cellular defense mechanisms, is intricately associated with the progression of UC, intestinal fibrosis, and CRC. Activation of Nrf2 activity is an effective therapeutic modality against oxidative stress-related diseases (8, 157). Recent studies have demonstrated both sides of Nrf2 in treating malignant tumors. It has been demonstrated through previous studies that the excessive activation of Nrf2 contributes significantly to malignant tumor transformation, treatment resistance, and unfavorable clinical outcomes. Inhibition of over-activated Nrf2 activity in tumor cells can exert anti-tumor effects by disrupting redox homeostasis, antagonizing tumor metabolism, and reversing drug resistance in various ways (15, 158, 159). Therefore, the study of the role of Nrf2 and the molecular mechanism of its activity is becoming a new hot topic.

The literature shows that many medicinal plants and phytochemicals, synthetic chemicals or inducers, and others, such as metformin and short-chain fatty acids, modulate the effects of Nrf2 on UC and UC-associated colorectal cancer by activating Nrf2-mediated antioxidant expression and attenuating NF-κB-associated inflammation (**Table 1**). Studies have shown that Nrf2 inhibits NF-κB, the most important mediator of UC inflammation, through several cellular and biochemical mechanisms (68, 160, 161). At its peak expression, Nrf2 attenuates the activity of NF-κB primarily by hampering the generation of ROS. The activation of Nrf2 reduces ROS levels, consequently inhibiting the production of NF-κB-dependent pro-inflammatory factors activation mediated by ROS (68). The maintenance of stable Nrf2 activation levels during the initial stages of UC is likely to enhance the intestinal environment, bolster the mucosal barrier, prevent the disruption of the colon, reduce ulceration and microbial metastasis, ultimately suppressing the disease activity index (DAI), restraining the progression of UC, and mitigating the likelihood of subsequent complications (162–164). Moreover, Nrf2 signaling modulates a multitude of genes implicated in redox regulation, protein degradation, DNA repair, xenobiotic metabolism, and apoptosis, which collectively impede the development of colorectal cancer associated with ulcerative colitis (55, 165, 166). Research indicates that in the early stages of various inflammatory disorders, maintaining constant levels of Nrf2 activators can inhibit progression, thereby preventing complications like fibrosis and cancer. However, in advanced cancers wherein Nrf2 expression is elevated, Nrf2 inhibitors may serve as efficacious therapeutic adjuvants that can significantly reduce radiotherapy resistance (3, 126). Nevertheless, further investigations are essential to illuminate the intricate role of Nrf2, along with developing novel drugs capable of modulating the Nrf2 pathway and potentiating its defensive effects.

**Table 1:** Studies the therapeutic impact of controlling the Nrf2 signaling pathway using medication on UC.

Compound	Optimal Doses (/kg Body Weight)	Model	Potential Mechanism	PMID
Ulcerative colitis				
Natural products				
			**Activating the Keap1/Nrf2/HO-1 pathway**	
Luteolin	50mg	DSS-induced acute colitis in C57BL/6 mice	Mitigation of colitis in murine models by stimulating the Nrf2 signaling pathway	27569028
Hyperoside	120mg	DSS-induced acute colitis in C57BL/6 mice	The Nrf2 signaling pathway activation mitigates colonic inflammation and diminishes apoptosis.	29162986
Gallic Acid	10mg	DSS-induced acute colitis in BALB/c mice	Stimulates or enhances the expression of Nrf2 and its downstream targets.	26251571
Procyanidin B2	30mg	DSS-induced acute colitis in C57BL/6 mice	Repress oxidative stress *via* Nrf2/ARE signaling	32940048
Caffeic Acid	251mg	DSS-induced acute colitis in ICR mice	Activating the Nrf-2/HO-1 pathway	34867926
Dieckol	15mg	DSS-induced acute colitis in C57BL/6 mice	The suppression of inflammatory signaling and activation of the Nrf2/HO-1 signaling pathway results in the mitigation of colitis.	33331035
Thymoquinone	40mg	DSS-induced acute colitis in C57BL/6 mice	Reducing inflammation through the Nrf2/Keap1 system	33051921
Sinomenine	100mg	DSS-induced acute colitis in C57BL/6 mice	The Nrf2/NQO 1 signaling pathway-mediated alleviation of colitis.	30106158
Matrine	1g	DSS-induced acute colitis in C57BL/6 mice	Activates/upregulates the expression of Nrf2 and its downstream targets	30284461
Berberine	40mg	DSS-induced acute colitis in Sprague-Dawley rats	Alleviation of colitis in rats through the Nrf2-dependent mechanisms	29891588
Rutaecarpine	80mg	DSS-induced acute colitis in C57BL/6 mice	Inhibition of KEAP1-NRF2 interaction and activation of NRF2	31874248
Imperatorin	60mg	TNBS-induced colitis in Sprague–Dawley rats	The modulation of the Nrf-2/ARE/HO-1 pathway in rats.	33098052
Astragalus polysaccharides	300mg	DSS-induced acute colitis in C57BL/6 mice	Activates the NRF2/HO-1 pathway	34562468
6-Shogaol	15mg	DSS-induced acute colitis in FVB/NJ mice	Induce Nrf2 and activate Nrf2 target genes in an Nrf2-dependent manner	28961808
Oligonol	50mg	DSS-induced colitis in C57BL/6 mice	Prevented the relapse of colitis through enhancing NRF2-mediated antioxidative defense mechanism	30149369
Alpinetin	100mg	DSS-induced acute colitis in C57BL/6 mice	Nrf2/HO-1 signaling pathways were found to be activated	29661352
Sulforaphane	15mg	Acid acetic solution induced colitis in Sprague–Dawley rats	Increase the expression of Nrf2 and HO-1	35754320
Crocin	20mg	Acid acetic solution induced colitis in Sprague–Dawley rats	Enhancement of Nrf2 and HO-1 signaling and down-regulation of caspase-3 activity	30530041
Quercetin	20mg	DSS-induced acute colitis in Wistar rats	Reduces the Nrf2 and HO-1 gene expression	35884960
PMID	22mg	DSS-induced acute colitis in ICR mice	Induces the activation of Nrf2/ARE pathway	25874026
Ruscogenins	2mg	TNBS-induced colitis in C57BL/6 mice	Activation of the Nrf2/HO1 signaling pathway	35308175
Apocynin	400mg	DSS-induced acute colitis in BALB/c mice	Anti-inflammatory mediators Nrf2 and HO-1 were activated	31141554
Hesperidin	40mg	DSS-induced acute colitis in C57BL/6 mice	Protect against intestinal inflammation via enhanced Nrf2 antioxidant pathway, increases the Treg population	30817082
Carnosic Acid	100mg	DSS-induced acute colitis in BALB/c mice	Modulation of the Keap1/Nrf2 pathway plays a preventive role in colitis.	28887507
Protocatechuic Acid	60mg	TNBS-induced colitis in C57BL/6 mice	Elevated expression of Nrf2 and antioxidant enzymes, coupled with reduced expression of proinflammatory cytokines	28300788
Ficus pandurata Hance	48g	DSS-induced acute colitis in C57BL/6 mice	Facilitation of colonic antioxidative stress attributes, accomplished through the elevation of T-SOD and GSH-Px levels and the augmentation of NRF2 and HO-1 expressions.	34966476
Moringa oleifera Lam	150mg	DSS-induced acute colitis in C57BL/6 mice	Upregulated GSTP1, an Nrf2-mediated phase II detoxifying enzyme	28922365
Atractylenolide III	20mg	TNBS-induced colitis in C57BL/6 mice	Regulating oxidative stress via the FPR1 and Nrf2 pathways	34156157
Pisum sativum L	600mg	DSS-induced acute colitis in C57BL/6 mice	The amelioration of DSS-induced colitis is accomplished by modulating the Keap1/Nrf2 pathway and gut microbiota.	34829046
Rhus chinensis Mill	600mg	DSS-induced acute colitis in C57BL/6 mice	Upregulation of the expression of Nrf2, NQO1 and HO-1	34494061
Crocus sativus	25mg	DSS-induced acute colitis in C57BL/6 and BALB/c mice	Activation of AhR-Nrf2-dependent pathways	34275090
Prunus mahaleb	1300mg	DSS-induced acute colitis in BALB/c mice	The activation of the Nrf2 pathway serves to potentiate mitochondrial oxidative metabolism.	31410984
Bruguiera gymnorrhiza	100mg	DSS-induced acute colitis in BALB/c mice	Mitigating inflammatory and oxidative states is achieved by activating the Keap1/Nrf2 signaling pathway.	32116661
Honokiol	40mg	DSS-induced acute colitis in C57BL/6 mice	Reduction of colitis through the inhibition of oxidative stress and inflammatory signaling.	36578522
Dendrobium officinale	200mg	DSS-induced acute colitis in BALB/c mice	Inhibition of TLR4 and the activation of Nrf2 signaling pathway	34363819
			**Regulating Nrf2/NF-kB signaling pathway**	
Puerarin	50mg	DSS-induced acute colitis in BALB/c mice	The alleviation of colitis in murine models via the activation of the Nrf2 pathway, coupled with the reduction of NF-κB	31981944
Pectolinarigenin	10mg	DSS-induced acute colitis in C57BL/6 mice	The regulation of the NF-κB/Nrf2 pathway diminishes colitis in murine models.	35931839
Wogonin	50mg	DSS-induced acute colitis in BALB/c mice	The modulation of the Nrf2 signaling pathway curtails TLR-4/NF-κB activation.	24901054
Sericic acid	50mg	DSS-induced acute colitis in C57BL/6 mice	The mitigation of colitis through the modulation of NF-κB and Nrf2 pathways.	36173058
Triptolide	0.02mg	DSS-induced acute colitis in C57BL/6 mice	The stimulation of the NRF2/HO-1 signaling pathway, coupled with the inhibition of the PDE4B/AKT/NF-κB pathway.	33240279
Asperuloside	500ug	DSS-induced chronic colitis in KM mice	Mitigating oxidative stress and inflammation in colitis through the modulation of the Nrf2/HO-1 and NF-κB pathways.	33974900
Dehydrocostus Lactone	15mg	DSS-induced acute colitis in ICR mice	The mitigation of colitis via the modulation of the Keap1-Nrf2 and IKKα/β-NF-κB signaling pathways.	35321327
Geniposide	40mg	DSS-induced acute colitis in ICR mice	The Nrf2/HO-1/NF-κB pathway-mediated abatement of colitis in murine models.	32787366
Epoxymicheliolide	5mg	DSS-induced acute colitis in ICR mice	The prevention of colitis is accomplished through the inhibition of the TAK1-NF-κB pathway and activation of the Keap1-NRF2 pathway.	36461599
Myristicin	150mg	Acid acetic solution induced colitis in Sprague–Dawley rats	The targeting of endoplasmic reticulum stress, Nrf-2/HO-1, and NF-κB signaling pathways.	36403646
Licochalcone A	80mg	DSS-induced colitis in C57BL/6 mice	Downregulate NF-κB pathway and upregulate Nrf2 pathway	29710547
Leonurine	30mg	DSS-induced acute colitis in C57BL/6 mice	Inhibited TLR4/NF-κB pathway and activated of Nrf2/HO-1 pathway	35253649
GB1a	100mg	DSS-induced acute colitis in C57BL/6 mice	Repression of NF-κB and activation of Nrf2 signaling pathway.	34557497
Diosmetin	50mg	DSS-induced acute colitis in C57BL/6 mice	Elevate the levels of Nrf2 and HO-1 while simultaneously diminishing the acetylated NF-κB and NF-κB ratio by activating the circ-Sirt1/Sirt1 axis.	34262136
Ulva pertusa	100mg	DNBS-induced colitis in CD1 mice	Mitigation of DNBS-induced colitis damage is achieved through the modulation of the NF-κB/Nrf2/SIRT1 signaling pathways.	35893393
Panax ginseng	0.3g	DSS-induced acute colitis in C57BL/6 mice	Concomitant inhibition of the MAPK/NF-κB signaling pathway and activation of autophagy and p62-Nrf2-Keap1 signaling pathways, ameliorating inflammation.	34648903
Rose odorata sweet var. gigantean	500mg	DSS-induced acute colitis in C57BL/6 mice	Regulating the Nrf2/NF-κB signaling pathways	35692958
Perilla frutescens	100mg	DSS-induced acute colitis in ICR mice	Inhibited the activation of both NF-κB and STAT3 and elevated the accumulation of Nrf2	28848431
D-pinitol	40mg	DSS-induced acute colitis in BALB/c mice	Activating Nrf2/ARE and PPAR-γ/NF-κB signaling pathways	33625409
Mesua assamica (King&Prain) Kosterm	200mg	DSS-induced colitis in C57BL/6 mice	Exacerbation of colitis is achieved through the inhibition of NF-κB/STAT3 signaling, and the activation of HO-1/Nrf2/SIRT1 pathways.	36195303
			**Activating the Nrf2/HO-1 pathway and suppresses NLRP3 inflammasome**	
Cardamonin	60 mg	DSS-induced acute colitis in C57BL/6 mice; TNBS-induced colitis in BALB/c mice	The AhR/Nrf2/NQO1 pathway mediated suppression of NLRP3 inflammasome activation.	30071202
Norisoboldine	40mg	TNBS-induced colitis in BALB/c mice	The modulation of AhR/Nrf2/ROS signaling pathway effectively suppresses NLRP3 inflammasome activation.	29576052
8-Oxypalmatine	50mg	DSS-induced acute colitis in BALB/c mice	The regulatory modulation of Nrf2 and NLRP3 inflammasome signaling leads to a superior anti-colitis effect.	35779424
Rosmarinic Acid	20mg	DSS-induced acute colitis in C57BL/6 mice	Modulation of NLRP3 inflammasome and reestablishment of the Nrf2/HO-1 signaling pathway	33530569
Toosendanin	1mg	DSS-induced acute colitis in C57BL/6 mice	Regulating NLRP3 inflammasome and Nrf2/HO-1 signaling	31520988
			**Other functional mechanisms**	
Gingerenone A	20mg	DSS-induced acute colitis in C57BL/6 mice	Amelioration of colitis via activating Nrf2-Gpx4 signaling pathway	36135333
Schisandrin B	10mg	DSS-induced acute colitis in C57BL/6 mice	Activation of AMPK/Nrf2 dependent signaling-ROS-induced mitochondrial damage	34628369
Sesamin	100mg	DSS-induced acute colitis in C57BL/6 mice	Through the activation of AKT and ERK signaling pathways, the activation of Nrf2-mediated protective responses against oxidative stress and inflammation is potentiated in colitis.	31534619
Aucklandia lappa Decne	1.82g	DSS-induced acute colitis in C57BL/6 mice	Colitis attenuation is attained by co-modulating MAPK and Nrf2/Hmox-1 signaling pathways.	35623504
Ziziphus spina-christi	400mg	Acid acetic solution induced colitis in Wistar rats	The induction of Nrf2 and HO-1 expression suppresses oxidative stress and p38 MAPK expression, resulting in the mitigation of colitis.	29518435
Chemical drugs				
			**Activating the Nrf2/HO-1 pathway**	
5-ASA	30mg	TNBS-induced colitis in Sprague-Dawley rats	Activates Nrf2-HO-1 pathway by covalently binding to Keap1	28473247
Telmisartan	7mg	DSS-induced colitis in Sprague-Dawley rats	Upregulated the gene expression of Nrf-2 and HO-1	31326516
Coenzyme Q10	100mg	Acid acetic solution induced colitis in Sprague-Dawley rats	Modulation of Nrf2/HO-1 and caspase-3 pathways	28050757
Miconazole	20mg	Acid acetic solution induced colitis in Sprague-Dawley rats	Activation of Nrf2-regulated cytoprotective expression	35205169
			Regulating Nrf2/NF-kB signaling pathway	
Metformin	500mg	LPS-induced colitis in C57BL/6 mice	Alleviated NF-κB phosphorylation, promoted Nrf2 nuclear translocation, and increased the expression of the antioxidative genes	29772687
Olmesartan	10mg	Acid acetic solution induced colitis in Wistar rats	Ameliorates colitis via modulating NFκB and Nrf-2/HO-1 signaling	30594690
Carbocisteine	500mg	Acid acetic solution induced colitis in Sprague-Dawley rats	A Modulator of Nrf2/HO-1 and NF-κB Interplay	35754464
Corynoline		DSS-induced colitis in BALB/c mice	Modulating the Nrf2/NF-κB pathway	35980837
			**Activating the Nrf2 pathway and suppresses NLRP3 inflammasome**	
Mycophenolate mofetil	50mg	Acid acetic solution induced colitis in Sprague-Dawley rats	Targeting Nrf-2 and NLRP3 inflammasome	33539910
Other				
			**Activating the Nrf2 pathway**	
Dimethyl fumarate	25mg	DSS-induced chronic colitis in C57BL/6 mice	Activation of Nrf2-mediated antioxidant and anti-inflammatory pathways	32344663
Maggot	1g	DSS-induced acute colitis in C57BL/6 mice	Alleviate inflammation and oxidative stress in colitis via the activation of Nrf2	31827675
FSGHF3	200mg	DSS-induced acute colitis in C57BL/6 mice	Exert a protective effect on colitis via the Nrf2 pathway	31825438
Gloeostereum incarnatum	4g	DSS-induced chronic colitis in C57BL/6 mice	Enhanced the expression levels of Nrf2	32945507
			**Regulating Nrf2/NF-kB signaling pathway**	
Sodium Butyrate	1g	DSS-induced acute colitis in C57BL/6 mice	Inhibiting oxidative stress and NF-κB/NLRP3 activation via COX-2/Nrf2/HO-1 activation and mitophagy	36867295
Maresin 1	0.3ug	DSS-induced colitis in Sprague-Dawley rats	Alleviates colitis by regulating NRF2 and TLR4/NF-kB signaling pathway	31780371
			**An inhibitor of the Keap1-Nrf2 protein-protein interaction**	
CPUY192018	10mg	DSS-induced acute colitis in C57BL/6 mice	An inhibitor of the Keap1-Nrf2 protein-protein interaction	27215610
Colon cancer				
			Regulating the Nrf2 pathway	
Procyanidin B2	30mg	AOM/DSS-induced colitis-associated colorectal cancer in C57BL/6 mice	The capacity to reduce Nrf2 degradation and increase nuclear translocation of Nrf2 is observed.	32940048
Resveratrol	200mg	AOM/DSS-induced colitis-associated colorectal cancer in C57BL/6 mice	The activation of Nrf2 signaling for preventing colorectal cancer development, facilitated by resveratrol, is contingent on the interplay between Nrf2 and Mpk-1.	30844440
Digitoflavone	5mg	AOM/DSS-induced colitis-associated colorectal cancer in C57BL/6 mice	Elevation of Nrf2 expression, accompanied by its nuclear translocation and downstream expression of Phase II antioxidant enzymes.	24602443
Theobroma cacao	100g	AOM/DSS-induced colitis-associated colorectal cancer in BALB/c mice	Activation of the Nrf2 system	25545372
Glucosinolates	100mg	AOM/DSS-induced colitis-associated colorectal cancer in BALB/c mice	The expression of various Nrf2 target genes is upregulated.	24714741
Crocin	200mg	AOM/DSS-induced colitis-associated colorectal cancer in ICR mice	The suppression of NF-κB and subsequent downregulation of TNF-α, IL-1β, IL-6 expression, alongside the elevation of Nrf2 expression.	23326291
Pterostilbene	250mg	AOM/DSS-induced colitis-associated colorectal cancer in BALB/c mice	Activation of Nrf2 serves to block inflammation and oxidative stress by inducing the expression of HO-1 and GR, hence inhibiting colon carcinogenesis induced by AOM.	21355597
Nobiletin	–	AOM/DSS-induced colitis-associated colorectal cancer in CD-1 mice	The downregulation of iNOS, upregulation of Nrf2-dependent enzymes, and profound modulation of crucial signaling proteins reduce cell cycle progression.	28107678
Wogonin	5mg	AOM/DSS-induced colitis-associated colorectal cancer in C57BL/6 mice	The modulation of Nrf2 activation and diminution of nuclear translocation of NF-κB is observed.	24901054
Cinnamaldehyde	–	AOM/DSS-induced colitis-associated colorectal cancer in C57BL/6 mice	Ubiquitination blockage is observed, concomitant with the upregulation of Nrf2 cytoprotective target genes and rise in cellular glutathione levels.	25712056
			Regulating the Nrf2 pathway and amplifying the HO-1 expression	
Tussilagone	5mg	AOM/DSS-induced colitis-associated colorectal cancer in BALB/c mice	The manifestation of antioxidant effects in suppressing tumor formation is characterized by amplified HO-1 expression.	32290483
AcEGCG	–	AOM/DSS-induced colitis-associated colorectal cancer in ICR mice	The amplification of HO-1 expression is facilitated through ERK1/2 signaling and Nrf2 acetylation.	22409325

- means that the drug dose is not mentioned. Bold text means grouped activators according to their functional mechanisms.

## 8 Conclusions and perspective

The Keap1/Nrf2 axis significantly influences the healthy development and maintenance of the gastrointestinal tract’s normal function. Given its significant association with UC and its dire complications, the Keap1/Nrf2 axis is a potential therapeutic target for preventing such ailments. In this review, we describe the structure and function of Nrf2 and its role in intestinal development while we elucidate the critical role of Nrf2 in UC and its complications. First, Nrf2 can alleviate intestinal injury and inflammation by controlling oxidative stress. Secondly, Nrf2 activation modulates the expression of tight junction proteins, consequently supporting and fortifying the integrity of the intestinal epithelial barrier. Thirdly, Nrf2 activation regulates intestinal immunity to varying degrees and influences the development of UC. Finally, Nrf2 is closely associated with intestinal fibrosis and colorectal cancer that occur in UC. In summary, comprehending the intricacies underlying the structure and functionality of Nrf2, developing drugs that target the molecule effectively, and meticulously regulating its activity at different stages of the disease progression could offer new and innovative hypotheses to tackle ulcerative colitis management.

## Author contributions

SP and LS wrote the draft of the manuscript. SP drew the figures. XY, LZ, KX, YX, LLZ, and JW contributed to the literature search and discussion. LS and HL designed and revised this manuscript. All authors contributed to the article and approved the submitted version.
